# How Wastewater Monitoring is Helping to Investigate a Hepatitis A Outbreak Linked to Oysters’ Consumption

**DOI:** 10.1007/s12560-026-09694-2

**Published:** 2026-05-13

**Authors:** F. S. Le Guyader, J. Figoni, J. Ollivier, A. Chaghouri, S. Parnaudeau, A. Nisavanh, C. Le Mennec, J. Normand, A. Deslandes, M. Martel, S. Erouart, A.-M. Roque-Afonso

**Affiliations:** 1 Ifremer, U. Microbiologie Aliment Santé Environnement, LSEM/RBE Nantes, BP 21105, 44311 Nantes Cedex 03, France; 2 Santé Publique France, Saint-Maurice, France; 3 Centre National de Référence Hépatite A, Université Paris-Saclay, Inserm U1993, AP-HP Hôpital Paul Brousse, Villejuif, France; 4 Ifremer Laboratoire Environnement Ressources de Normandie, Port en Bessin, France; 5 Agence Régionale de Santé Normandie, Caen, France

**Keywords:** Hepatitis A virus, Outbreak, Shellfish, Sewage, One-health approach

## Abstract

In April 2024, two cases of hepatitis A (HA) were linked to the consumption of oysters produced in the same region, which triggered a multidisciplinary study. A network was quickly set up in a one-health approach including epidemiological and clinical data, as well as analysis of locally collected shellfish and wastewater samples. By the end of the outbreak, up to 17 HA cases met the case definition: all shared a same genotype IA strain and have eaten oysters. The prompt response enabled the identification of oyster samples positive for the HAV genome at the start of the monitoring period; these oyster samples subsequently tested negative during the following two months of the sampling campaign. Monitoring three nearby wastewater treatment plants revealed that the contamination was limited to a single area, allowing for more effective monitoring of shellfish. None of the identified cases lived in the area served by the treatment plant, and no clinical cases were detected among the local population, either before or after the outbreak. The decline of HAV concentration in sewage signed the end of the outbreak. While predicting contamination of shellfish growing areas is complicated, sewage surveillance can help establishing an alert system to prevent shellfish contamination, which is an important issue for silent diseases.

Shellfish, filtering large volumes of water for their physiological activities and being cultivated in coastal areas, are sensitive to contamination by human sewage. Since often associated with organic matter, a potential source of nutrients, viruses present in the population are filtered by shellfish. This is particularly concerning for enteric viruses, as they are highly resistant to wastewater treatment and appear to retain their infectivity in both wastewater and seawater (Carmona-Vicente et al., 2024; Desdouits et al., 2022; Kosgei, et al., 2025). Consequently, shellfish, particularly oysters eaten raw, are frequently implicated in foodborne outbreaks, with norovirus (NoV) infections being the most commonly reported (Flint et al., 2025; Teunis et al., 2020; Yu et al., 2015). Hepatitis A virus (HAV), a member of the *Picornaviridae* family, is a highly resistant non-enveloped RNA virus, also transmitted via the fecal–oral route (Pinto et al., 2010). The epidemiology of HAV is linked to socio-economic indicators, with greater circulation in developing countries, and a clustering of HAV strains by geographical regions (Mackowiak et al., 1976; Nainan et al., 2006). A large proportion of infections are asymptomatic, particularly among young children, and are therefore not captured by mandatory case reporting systems, which exist in all EU countries, including France. The transmission of HAV through shellfish is also a cause of concern and HAV outbreaks linked to the consumption of these products were among the first to be described in the literature (Halliday et al., 1991; Mackowiak et al., 1976; Pinto et al., 2010).

The difficulty in investigating HAV-associated outbreaks lies in its long incubation period of approximately four weeks, which complicates food consumption investigations and microbiological analysis of food, especially fresh food such as shellfish. In France, there is little published data on the management of such epidemics. However, several situations have been investigated in the past decades. In 1999, an outbreak of 33 cases, occurring in western France (Brittany) was linked to oyster and shellfish consumption in a case–control study (unpublished data). In 2007, 111 cases were found in that same region, all of which were infected with a common viral strain. Of the 89 interviewed cases 81% had eaten raw shellfish and 72% had eaten oysters. The collection of environmental samples (shellfish, sediments, wastewater) carried out late (two to three months after the presumed date of contamination of the oysters) did not reveal any viral sequences (Guillois-Becel et al., 2009). These events and the repeated detection of HAV in oysters and wastewater, whether linked to human cases or not, led the French Agency for Food, Environmental and Occupational Health & SafetyFrench to issue recommendations in 2010 aimed at securing sewage and sanitation systems and improving surveillance in areas close to shellfish farms (Denis et al., 2010). Nine years later, on a small French island, nine hepatitis A cases were notified, seven of whom had collected shellfish from the shore. It couldn’t be determined if shellfish consumption was the cause of infection, but to limit the spread of the disease, an environmental survey was conducted, as part of the management measures. HAV RNA was detected for two months in wastewater samples taken at the inlet of wastewater treatment plants (raw wastewater), but no signal was detected in treated wastewater samples or in the 19 shellfish samples analysed (Unpublished data; Schaeffer et al., 2023). These data highlight the difficulty of detecting or predicting contamination in shellfish growing areas.

In April 2024, two cases of hepatitis A were notified in two different French regions, both had consumed oysters originating from the same location. The identified production area was located on the north coast of France (Fig. 1), with approximately 4,800 tonnes of oysters produced each year over an area of 190 hectares. An integrated investigation was rapidly launched to propose measures to contain the outbreak. First, as soon as the suspected role of shellfish in the outbreak was raised, the competent authority advised producers in the suspected area to extend the purification period or to refrain from marketing their products. Subsequently, after the presence of the virus was confirmed in the local wastewater and in samples of shellfish, the production area was closed. A common genotype IA HAV strain (GenBank n° PX830474) was identified in clinical samples by the National Reference Centre for HAV (NRC-HAV). All Regional Health agencies have been asked to ensure that HAV IgM-positive serum samples positive for anti-HAV IgM are sent to the NRC-HAV for sequencing, and to report HAV cases having consumed oysters to food safety authorities so that they could investigate the traceability of the oysters. Environmental samples (shellfish and sewage) were collected to detect the presence of viral contamination. We describe in this manuscript, the investigations carried out and the lessons learned from this event.

**Fig. 1:**
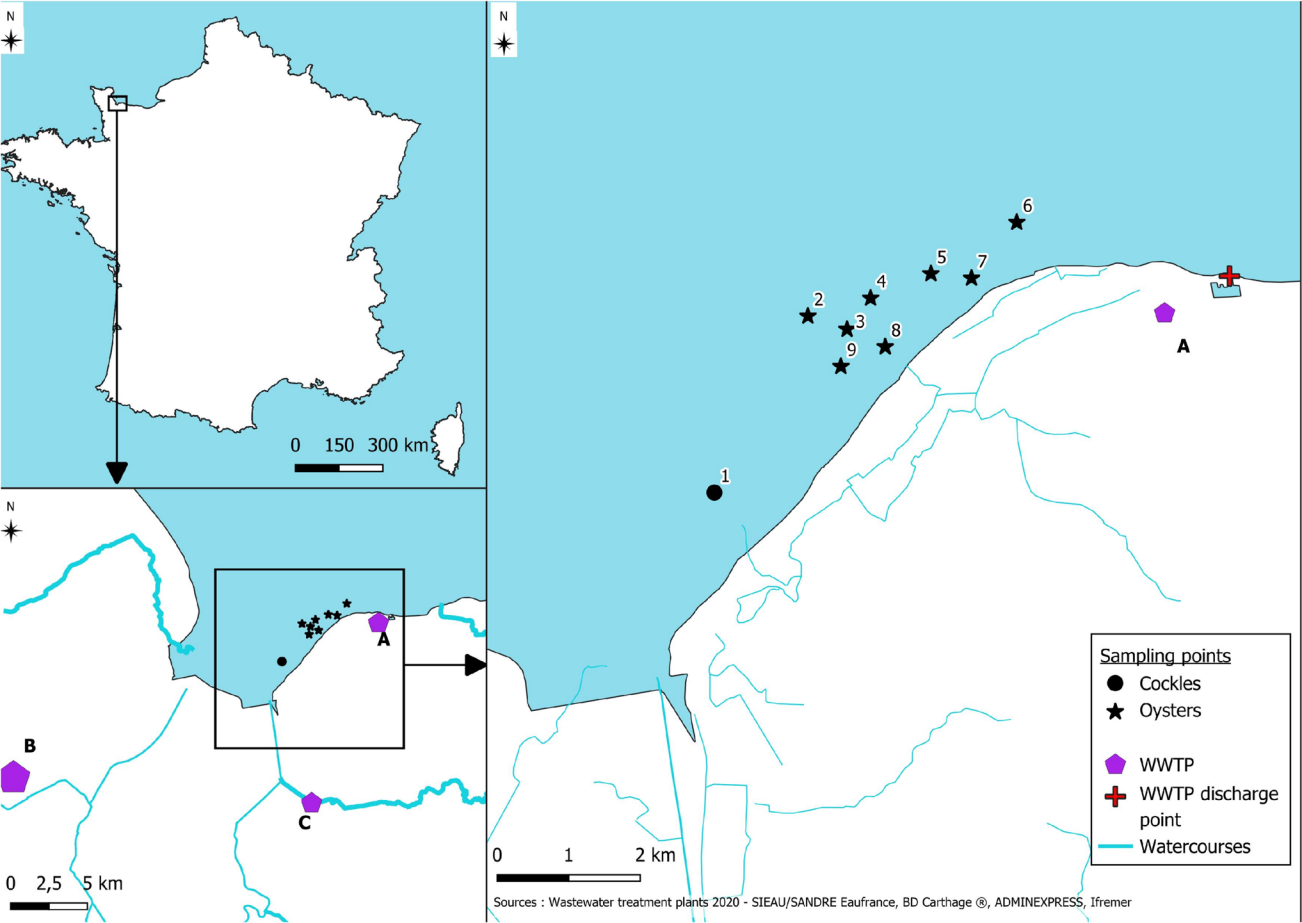
Localisation of the producing area and sampling points. Legend: The study area was in northern coast of France (upper left map), more precisely in Normandy area). Three sewage treatments plants were identified (lower left map), the pentagon size is proportional to the WWTP capacity. The localization of the nine shellfish points monitored are reported in the right map

## Method

### Epidemiological Investigation

In France, HAV surveillance is based on mandatory reporting of clinical cases. Notifications are submitted to regional health agencies and centralized for surveillance purposes by the national agency of public health (Santé Publique France). The consumption of shellfish and oysters is included in the notification form, but food investigations are only conducted when a common source of infection is suspected for at least 2 cases.

The case definition was a HAV infection with the epidemic strain, and a reported consumption of oysters.

### Environmental Investigations

For the purpose of this investigation, up to nine sampling points were monitored for HAV RNA. This production area is dedicated to oysters (*Magallana gigas*), except sampling point 1 producing cockles (*Cerastoderma edule*). Based on *Escherichia coli* monitoring in shellfish meat (flesh and intervalvular liquid) (European regulation 2073/2005), sampling point 1 is classified as “degraded” (class C) with heat treatment required before commercialisation, points 2, 3, 4, 5 as “low microbial quality” (class B) with purification needed before marketing, and only the production of point 6 can be put on the market directly (class A) (European regulation 2019/627). Based on data provided by infected consumers, samples were collected from three producers’ storage facilities, providing fairly broad spatial coverage (points 7, 8, 9). Six sampling points (Point 1, 2, 6, 7, 8 and 9) were collected four times, while three sampling points (points 3, 4, 5) were collected three times over seven weeks. All these nine sampling points were collected every two weeks due to the tides and used for HAV detection. One sample was constituted by at least 15 individuals (ISO 15216-1, 2017). In order to facilitate the assessment of potential viral contamination within the environment, NoV, the causative agent of gastroenteritis in humans and frequently detected in sewage, was also investigated.

Three wastewater treatment plants near the farming areas were identified (WWTP A, B, C) (Fig. 1). All three WWTP were using activated sludges as treatment with no tertiary treatment. Plants A and C collected sewage from communities of 7,000 and 6,000 inhabitants respectively, while plant B received sewage form 66,670 inhabitants. Samples (500 mL) were collected using 24-h samplers (composite sample).

Samples (shellfish and water) were sent to the laboratory within 48-h in cold conditions and immediately treated.

### Shellfish Sample Processing

Shellfish were shucked and the digestive tissues (DT) from 10 life individuals were dissected, carefully chopped, and homogenized before distribution into 2 g aliquots kept frozen (≤ − 20 °C) until analysis. The ISO 15216-1 method was applied with the addition of mengovirus (10^6^ TCID_50_) (kindly provided by A. Bosch, University of Barcelona, Spain) as a process control (ISO 15216-1, 2017). Viruses were eluted from DT using proteinase K method (ISO 15216-1, 2017).

### Wastewater Sample Processing

A 500 mL water sample was homogenized by moderate shaking, and a subsample of 11 mL was ultracentrifugated for 1 h at 100,000×*g*. The pellet was resuspended in 500 μL of Phosphate-Buffered Saline (PBS) and used for nucleic acid extraction (Wurtzer et al., 2020). No process control was added.

### Nucleic Acid Extractions and RT-PCR

Nucleic acids (NAs) were subsequently extracted by using the NucliSens reagents and the NucliSens miniMAG purification system (bioMérieux, France) following the manufacturer’s instructions, with 2 mL of lysis buffer, 50 μL magnetic silica and eluted in 100 μL elution buffer. For wastewater samples, extracted NAs were further purified using the OneStep PCR inhibitor removal kit (Zymo Research, USA), following the manufacturer’s instructions.

All amplifications were performed using the UltraSense One-Step quantitative RT-qPCR system under triplicate of 5 µL of undiluted NA (Life Technologies, France). All primers and probes for mengovirus, HAV (HAV68, HAV240 and HAV150), NoV (GI: QNIF4, NV1LCR, NVGG1p and GII: QNIF2, COG2R QNIFs) were as described (ISO 15216-1, 2017). Standard curves based on double stranded DNA (gBlocks, IDT USA) corresponding to nucleotides 80 to 240 for HAV pHM175 (Genbank M14707)*,* nucleotides 4484 to 5668 of the GI.1 Norwalk virus (Genbank M87661) or nucleotides 4217 to 5355 of the GII.4 Houston virus (Genbank EU310927), were included in each run, each concentration was tested in duplicate. All precautions were taken to prevent contaminations; negative extraction and amplification controls were included in each amplification series and filter tips and dedicated rooms were used. For each sample, the mean C_T_ value was calculated based on the 3 C_T_ values obtained. If the difference in the Ct values of the replicates exceeded 3 Ct, the test was invalidated and repeated. For the quantification of NoV, the genome copies (GC) concentration was calculated from the mean C_T_ value and the standard curve based on the volume of sample analysed, and expressed in GC per gram of DT for shellfish samples, with a limit of detection (LOD) of 14 GC/g for NoV GI and 16 GC/g for NoV GII, or per litre for wastewater samples, with a LOD of 10^3^ GC/L for the two genogroups (theoretical calculation). For the detection of HAV in shellfish, according to the ISO recommendation, only qualitative detection was performed (ISO 15216-1, 2017), with a LOD of 198 GC/g. In WWTP samples, HAV was quantified using the mean C_T_ value and the standard curve, with a LOD of 10^3^ GC/L (Wurtzer et al., 2020).

### HAV Sequencing

A fragment of 511 nucleotides encompassing the VP1/2A junction of the HAV genome was amplified as described (Desbois et al., 2010). VP1/2A amplicons were sequenced on both strands using the BigDye Terminator v3.1 Cycle Sequencing Kit (Thermofisher Scientific) on a SeqStudio Genetic Analyzer. Phylogenetic reconstruction was performed in MEGA11 (https://www.megasoftware.net/) using the neighbor-joining algorithm method, with evolutionary distances calculated using the Kimura two- parameter model, and bootstrap values determined from 1,000 bootstrap resamplings of the original data.

## Results

### Clinical Cases and Epidemiological Data

**Fig. 2:**
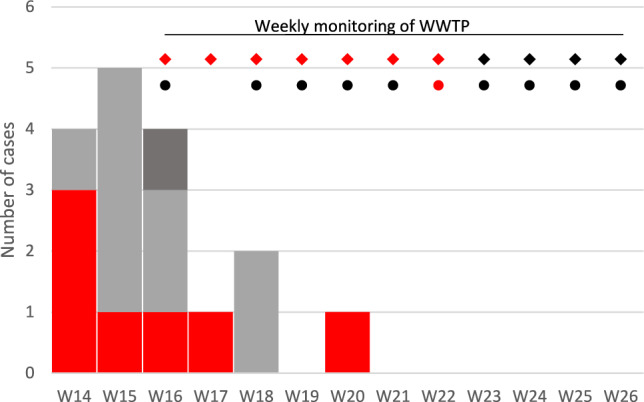
Weekly case incidence. The number of cases meeting the definition (epidemic strain and oyster consumption) is indicated by week of symptom onset or diagnosis. Red indicates oysters that could be traced to the implicated production area; grey indicates cases where traceability could not be established, and dark grey a case where traceability pointed to a different geographic area (see text for details). Wastewater treatment plants (WWTPs) were monitored from week 16 to week 26 (◆ WWTP A, ● WWTP C, WWTP B has always yielded undetectable results and does not appear on the graph). A black mark indicates a negative HAV result; a red mark indicates a positive result. None of the cases resided in the area served by the monitored WWTPs

As of week 27, (July 2nd), 17 cases of hepatitis A meeting the case definition, 7 women and 10 men aged 37 to 73 years, with symptoms’ onset between week 14 (April 1st) and week 20 (May 2nd) were identified. Of them, 13 were hospitalized but did not experience severe complications. In one case, traceability investigations linked the oysters consumed to a production area different to the one initially identified, suggesting another mode of contamination, in the absence of any alerts concerning the production area in question. In seven cases, oysters consumed between late February (week 9) and late April May (week 17) were traced back to the identified area, and in nine cases, the oyster traceability investigation was unsuccessful: epicurve for these 17 cases is shown Fig. 2. These patients had no other link between them than the consumption of oysters. None of the cases resided in an area close to the oyster production; only four of them resided in the same administrative region.

### Shellfish Samples

A total of 28 oyster and 4 cockle samples were collected from week 15 to week 21 (Table 1). All quality controls, such as extraction efficiency and absence of inhibitors, were valid except for one sample, for which despite repeated analysis the extraction efficiency was not valid according to the ISO method recommendations (ISO 15216-1, 2017). This sample collected on April 22 (W17) on point #5 was reported as uninterpretable.

**Table 1:** Detection of hepatitis A virus and norovirus in shellfish samples

Date	Point	Week 15		Week 17		Week 19		Week 21	
		HAV	NoV	HAV	NoV	HAV	NoV	HAV	NoV
Monitoring points	1	< lod	76	< lod	< lod	< lod	< lod	< lod	< lod
	2	< lod	< ld	POS	53	< lod	< lod	< lod	< lod
	3			< lod	< lod	< lod	< lod	< lod	< lod
	4			< lod	< lod	< lod	99	< lod	< lod
	5			unint	< lod	< lod	< lod	< lod	< lod
	6	POS	< ld	< lod	< lod	< lod	75	< lod	104
Produceur Fields	7	< lod	< ld	< lod	< lod	< lod	< lod	< lod	< lod
	8	POS	*	< lod	73	< lod	< lod	< lod	< lod
	9	< lod	77	< lod	< lod	< lod	78		

Hepatitis A virus (HAV) detection was performed following ISO 15216-2 method on a qualitative approach and expressed as positive (POS) sample or below the limit of detection (lod) (198 GC/g of DT). *: all nucleic acids extracted from the sample collected on April 10 were used in an attempt to sequence HAV, preventing NoV detection. For norovirus (NoV), the ISO 15216-1 quantitative method was used. Results are expressed as genomic copies/g (GC) of digestive tissues (DT) (both genogroups added), or below the limit of detection (< lod) (14 GC/g of DT for NoV GI and 16 GC/g of DT for NoV GII). Empty case means that no sample was collected. Samples collected in point 1 were cockles, all other samples were oysters. One oyster sample cannot be analyzed as the recovery efficiency was not correct (uninterpretable)

We detected HAV RNA in week 15 and week 17. In week 15, HAV RNA-positive samples came from sampling point #6 of the *E. coli* monitoring sampling point and from point #8 of a producer field. The PCR signals were weak with only one PCR replicate being positive among the three for both samples. On that date, NoV RNA was also detected at points #1 and #9. In week 17, HAV RNA was weakly detected at point #2, and NoV RNA was detected at point #2 and #8. Samples taken over the following weeks tested negative for HAV RNA, but four of them tested positive for NoV RNA. Samples collected in weeks 19 and 21 tested negative for HAV RNA, but four of them tested positive for NoV RNA.

### Sewage Samples

Raw sewage samples were collected weekly from week 16 to week 26, 11 from WWTP A, 10 from B, and 10 from C (Table 2). Comparable concentrations of NoV were detected in the raw sewage from the three treatment plants. HAV was detected in WWTP A from week 16 to week 22. Raw wastewater samples taken from treatment plants B and C were found to be below LOD, except for one sample taken on week 22 from treatment plant C (Table 2 and Fig. 2).

**Table 2:** Detection of hepatitis A and norovirus in sewage samples collected from three local sewage treatment plant

Week	WWTP A				WWTP B		WWTP C	
	HAV		NoV		HAV	NoV	HAV	NoV
	in	out	in	out	in	in	in	in
16	9.56 × 10^6^	2.17 × 10^4^	4.65 × 10^4^	1.05 × 10^4^			< lod	7.41 × 10^5^
17	2.10 × 10^6^	3.11 × 10^3^	4.43 × 10^4^	3.01 × 10^4^	< lod*	8.74 × 10^4^		
18	4.21 × 10^5^		1.23 × 10^6^		< lod	4.00 × 10^5^	< lod	2.60 × 10^6^
19	8.21 × 10^4^		7.01 × 10^4^		< lod	1.01 × 10^5^	< lod	9.88 × 10^5^
20	4.95 × 10^4^		2.05 × 10^6^		< lod	8.23 × 10^5^	< lod	9.21 × 10^5^
21	3.47 × 10^4^	< lod	7.98 × 10^5^	8.26 × 10^3^	< lod	1.30 × 10^6^	< lod	2.13 × 10^6^
22	5.30 × 10^3^	< lod	9.56 × 10^5^	2.50 × 10^3^	< lod	2.65 × 10^5^	7.94 × 10^3^	2.67 × 10^5^
23	< lod	< lod	5.46 × 10^6^	6.15 × 10^3^	< lod*	2.75 × 10^5^	< lod	8.78 × 10^6^
24	< lod	< lod	2.03 × 10^6^	3.42 × 10^4^	< lod*	7.81 × 10^5^	< lod	3.30 × 10^6^
25	< lod		2.22 × 10^5^		< lod	1.72 × 10^5^	< lod	1.67 × 10^6^
26	< lod		1.74 × 10^5^		< lod	3.43 × 10^5^	< lod	2.60 × 10^6^

Hepatitis A (HAV) and norovirus (NoV) were detected and quantified using real time PCR and expressed as genomic copies (GC) per liter (GI and GII cumulated for NoV), or reported as below the theoretical limit of detection (< lod) estimated to be 10^3^ GC/L for raw sewage (in) or treated sewage (out). For WWTP B and C no treated sewage sample was collected except on few occasions (*), and these were all below lod for HAV. Empty case mean that no sample was collected

Treated sewage collected at WWTP A tested positive for HAV RNA in weeks 16 and 17, with concentrations approximately 1,000 times lower than those observed in raw wastewater (Table 2). NoV concentrations in the treated water samples were also lower than in the raw wastewater, but the decrease was less significant than for HAV, particularly for the first two samples (Table 2). Three samples of treated water taken from WWTP B (week 17, 23, 24) did not yield any positive results for HAV (as indicated by * in Table 2).

### Sequence Comparison

**Fig. 3:**
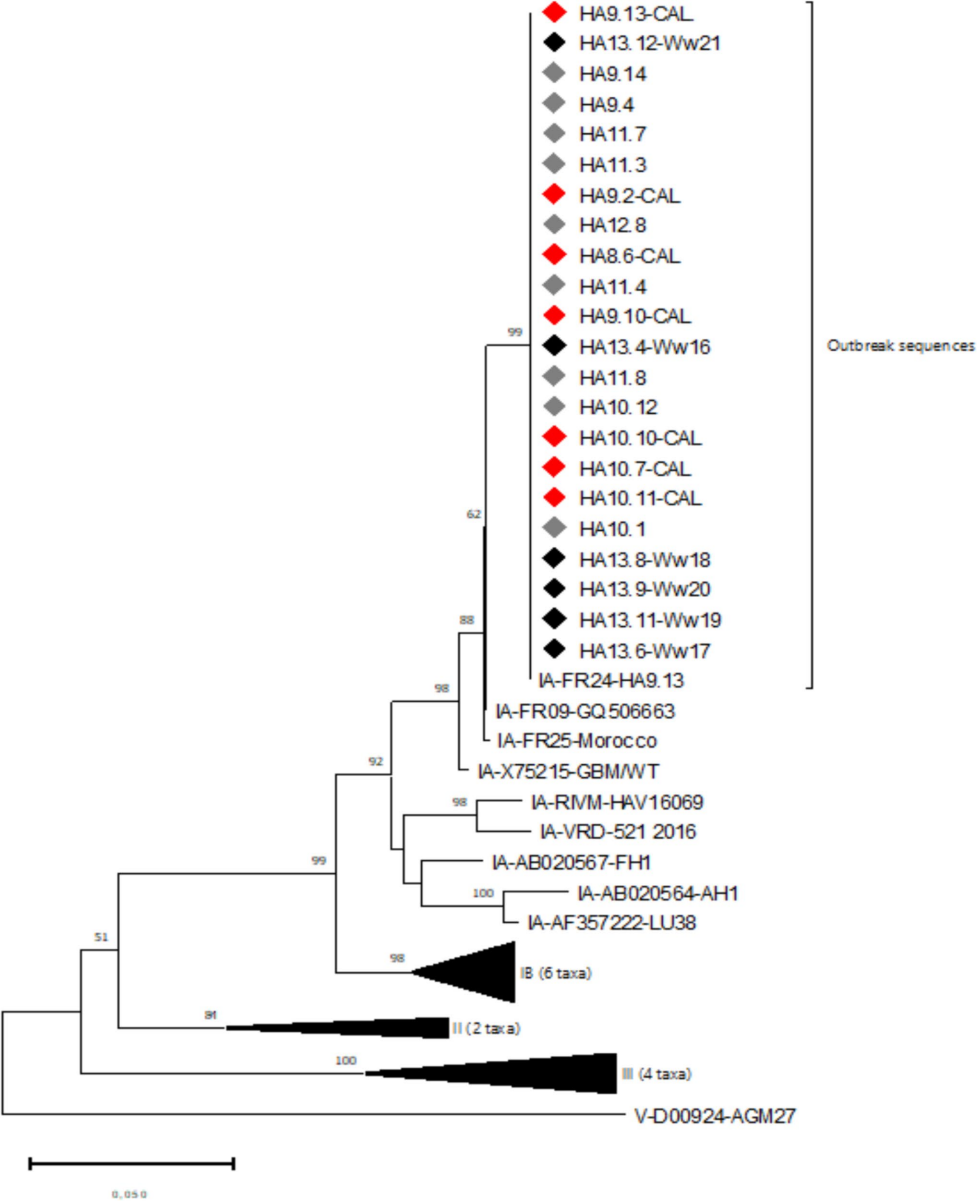
Phylogenetic analysis of HAV sequences from clinical cases and environmental sample. The evolutionary history was inferred using the Neighbor-Joining method from evolutionary distances computed using the Kimura 2-parameter method in MEGA11. The percentage of replicate trees in which the associated taxa clustered together in the bootstrap test (500 replicates) are shown above the branches. There was a total of 444 positions in the final dataset (♦ wastewater; ♦ Red symbol Epidemic strain with consumption of oysters traced back to the producing area; ♦ grey symbol Epidemic strain with consumption of oysters of unknown origin)

We obtained HAV nucleotide sequences from wastewater samples collected between week 16 and 21. These six sequences were homologous to those of the clinical cases (strain FR24-HA9.13), as shown on the phylogenetic tree (Fig. 3).

This sequence was present in the Reference Centre database but had only been found sporadically until this episode. In 2024, however, this sequence was identified throughout France, mainly in patients sampled in the second trimester, regardless of oyster consumption. It was no longer found in 2025.

### Other Information

We found no specific events that could have affected the operation of the treatment plant A during the presumed period of contamination of the oyster beds (week 8 to 13), such as heavy rainfall, sewer overflows or pipe problems. The fact that 25% of shellfish samples tested positive for NoV suggests that the effectiveness of treatment plant A in removing viral particles is limited, or that there are problems with the sewer system. This is a high percentage for this time of year, in line with the microbial quality of the producing area (EFSA, 2019; Ollivier et al., 2022).

## Discussion

In this paper we report on a joint investigation into an outbreak of hepatitis A linked to the consumption of oysters, as well as the measures taken to prevent further contamination through the assessment of the potentially contaminated area, based on monitoring of wastewater treatment plants, and the analysis of shellfish samples. At the same time, recommendations were issued to producers to strengthen their purification measures or refrain from marketing their products until the area is closed. Rapid information sharing allowed the detection of a HAV PCR signal in some oyster samples, but unfortunately it was too weak for sequencing. Investigating hepatitis A outbreaks is always complicated due to the long incubation period, which makes it difficult to identify the source of the infection. The second challenge is then to trace back the suspected food item and, above all, the batch that was consumed. This proves even more complicated when the suspected food is shellfish. Firstly, because a recent study revealed that French people living in coastal areas regularly consume shellfish, which are considered as a source of nutrients and a cultural food, particularly during winter and spring months (Lunghi et al., 2023). Regular consumers typically buy their oysters from a variety of producers, depending on where they live and the day of purchase. After a few weeks, they may have trouble remembering who the producer was. Secondly, shellfish, and especially oysters, are consumed fresh, so it is unlikely that the same batch will still be available two months after it was placed on the market. Direct-to-consumer sales are usually easier to trace. This is especially true when the farm has a low production. Although a label accompanies the product throughout the marketing process and identifies all batches of shellfish, precise traceability of oysters is still complicated. This is because, during production, oysters may be moved from one production area to another, or, when ready to eat, they can be sold in different areas of France. This may explain why for nine cases the oyster traceability failed. Additionally, the region where oysters are produced is also popular tourist destination for spring holidays, attracting visitors from all over the country and abroad. Aware of these challenges, we examined all cases of hepatitis A infected with the identified strain and who had consumed oysters throughout France.

Building on our experience gained during previous events, we quickly established a network to exchange information, coordinate sampling and share the results of epidemiological observations, clinical data and environmental sample analysis, as part of an integrated surveillance in a One Health approach. Rapidly we confirmed that the epidemic strain was detected in the nearby WWTP, making it likely that the production area had been contaminated by these effluents or by direct input of raw sewage due to a broken pipe or any other accidents on the network. However, with only preliminary environmental analyses and epidemiological data, it was unclear whether this production area was the only one to be contaminated. In order to prevent the sale of contaminated oysters, a sampling strategy was developed to assess the extent of potentially affected production areas. Wastewater samples were collected from different treatment plants to determine whether the contamination affected a larger production area or not. This approach confirmed that the contamination was limited to a single area, as only the first WWTP tested positive for the virus, allowing us to focus our analysis on shellfish collected on the area possibly contaminated by discharges from the incriminated WWTP. The decline of HAV concentrations in sewage was associated with the end of the event and the absence of further notified cases.

Unfortunately, no systematic testing is carried out to detect HAV in wastewater. Consequently, apart from clinical cases reported by medical doctors, no data is available regarding the virus prevalence in the population. The first cases developed symptoms during week 14, and, as expected, they had eaten oysters in weeks 8 to 10. These oysters were therefore likely contaminated by effluent from the wastewater treatment plant during the first weeks of 2024 or direct input of raw sewage into the coastal environment, but unfortunately no monitoring was being conducted at that time. This contamination likely lasted several months, since HAV was detectable at the plant’s outflow at least until week 17. However, despite active research among doctors and laboratories, no clinical cases were identified in the small town served by this WWTP in the first six months of 2024. Asymptomatic excretion by one or more residents—asymptomatic infections being common for hepatitis A (Abutaleb et al., 2020; Pinto et al., 2010)—or excretion by tourists visiting the region is likely to have caused the initial contamination. Subsequently, asymptomatic or unreported infections in residents were most likely responsible for the prolonged detection of HAV in wastewater. As France has a low endemic rate of hepatitis A and high standards of hygiene, most cases of hepatitis A are linked to imported strains, with little secondary transmission occurring in connection with these cases. Although microbiological surveillance of case strains in France is not exhaustive, it provides a fairly accurate picture of the actual viral diversity. The strain involved was not common in France prior to this epidemic, with only one case identified in southern France in December 2023 and another case in the Paris region in March 2024, with no direct link having been established to the oyster farming area, or the small town served by the WWTP in question, but other patients, whether asymptomatic or unreported, may have been in that town. As this strain was identified across France in a total of 34 patients in 2024, our case definition was restricted to the 17 patients who had consumed oysters. The other patients may have been secondary cases linked to asymptomatic individuals who had consumed oysters or may simply have travelled to the area where the epidemic strain is endemic.

Since 1973, several hepatitis A outbreaks have been reported that were linked to shellfish consumption. However, it is not known exactly how the shellfish became contaminated in the producing area (Desenclos et al., 1991; Goh et al., 1984; Mackowiak et al., 1976; Sanchez et al., 2002; Shieh et al., 2007). Following a HAV outbreak linked to mussel consumption in the Netherlands, a point-by-point investigation allowed to trace back the most likely source of contamination in the country of mussel production in the United Kingdom (Boxman et al., 2016). In this study, a patient infected abroad while on holiday and who lived near the mussel production area was identified as the first case. In the Netherlands, a second study was conducted to assess the prevalence of HAV in commercially available shellfish. Only one sample out of 392 mussel analysed and none out of 228 oyster samples tested positive for HAV, confirming that the prevalence of HAV contamination in European shellfish is low (Dirks et al., 2021). As HAV is of human origin, shellfish contamination is linked to the prevalence in the population. In Italy after a large outbreak in the Puglia (south Italy), a large-scale vaccination program was implemented (Chironna et al., 2012). Eleven years after, the incidence rate in the local population had become very low and all tested mussel samples produced locally were found negative for HAV (Chironna et al., 2012). More recently, in the same country, after an HAV contamination event, 352 shellfish samples were collected over 6 months from different production area. A total of 77 samples tested positive for HAV, all collected during the first three months of the study (Suffredini et al., 2017). In Brazil, as part of a study to evaluate the prevalence of human enteric viruses in two areas of shellfish production, up to 14% of mussel samples collected in the Rio de Janeiro area were detected contaminated with HAV while all shellfish samples collected in the Santa Catarina area tested negative (dos Santos et al., 2025).

To prevent hepatitis A outbreaks linked to shellfish consumption, various scenarios should be implemented, such as regular monitoring of shellfish contamination, the establishment of alert systems following the identification of clinical cases in coastal areas or upstream wastewater monitoring. However, even for norovirus—the pathogen most frequently implicated in shellfish-related outbreaks in Europe—there is still no regular monitoring of contamination in shellfish. Furthermore, with regard to HAV, the first two scenarios may be difficult to implement and of limited effectiveness partly because of the cost of sampling and testing shellfish, and given the high percentage of asymptomatic cases and the possibility that infected tourists may visit the area (Thebault et al., 2012). By contrast, as documented during the COVID-19 pandemia, wastewater surveillance is easy to implement, and can alert us to possible contamination of shellfish as well as being a useful complement to active surveillance of the disease, as suggested in France, Argentina, USA, Canada (Bisseux et al., 2018; Chettleburgh et al., 2025; Fantilli et al., 2023; Zulli et al., 2024). In Canada, HAV was detected in sewage before the identification of clinical cases (Chettleburgh et al., 2025). In Israel, the clear demonstration of the relationship between identified clinical cases and viral genomic detection in sewage add arguments to implement sewage surveillance for this virus (Gozlan, et al., 2024). In addition, in Israel and France, there may be positive sewage samples with no clinical cases reported, highlighting the importance of sewage surveillance for silent diseases (Bisseux et al., 2018; Gozlan et al., 2024).

Since the COVID19 pandemia, wastewater-based molecular epidemiology has attracted considerable interest due to its ability to detect the emergence of new strains or an increase in cases within the population (Wurtzer et al., 2020). When searching for a human virus such as HAV in coastal areas, there are various potential entry points, such as a damaged pipe, a malfunction at a pumping station, or inadequate wastewater treatment. In this study, we used wastewater analysis to confirm the presence of the virus in the local population, but above all to adapt our sampling strategy for shellfish. Questions remain about the impact of climatic events such as flood or drought, which are expected to be more frequent in the future, on the detection rate of HAV in wastewater, on how to determine the number of infected cases based on viral concentrations in water, and on the persistence of infectious viral particles in wastewater plants, as HAV is highly resistant to different physical treatment, staying infectious in different types of water or after freezing and cooking (Kevill et al., 2025; Pinto et al., 2009). Despite these limitations, the implementation of wastewater-based surveillance for HAV in upstream wastewater treatment plants could help to develop a preventive alert system to avoid shellfish contamination.

## Data Availability

No datasets were generated or analysed during the current study.
